# Evaluation of the Versant CT/GC DNA 1.0 Assay (kPCR) for the Detection of Extra-Genital *Chlamydia trachomatis* and *Neisseria gonorrhoeae* Infections

**DOI:** 10.1371/journal.pone.0120979

**Published:** 2015-03-23

**Authors:** Antonella Marangoni, Claudio Foschi, Paola Nardini, Monica Compri, Roberto Cevenini

**Affiliations:** Microbiology, Experimental Diagnostic and Specialty Department (DIMES), University of Bologna, Bologna, Italy; Xavier Bichat Medical School, INSERM-CNRS - Université Paris Diderot, FRANCE

## Abstract

Screening for extra-genital *Chlamydia trachomatis* and *Neisseria gonorrhoeae* infections is a crucial component for sexually transmitted diseases management, even if at present days no commercial methods have been approved for use on pharyngeal and rectal specimens by the US FDA or have received the conformity CE marking. Here we report the analytical sensitivities of the Versant CT/GC 1.0 assay (Siemens Healthcare Diagnostics, Tarrytown, NY, USA) on rectal and pharyngeal swabs, and an evaluation about the suitability for this assay with two widely used swab collection devices (E-Swab and eNAT, Copan, Brescia, Italy). The limits of detection for rectal and pharyngeal specimens with the Versant assay were 10 copies/ml and 1.0 copies/ml, for *C*. *trachomatis* and *N*. *gonorrhoeae*, respectively. False positive results due to the presence of non-gonococcal Neisseria species were excluded when clinical rectal and pharyngeal samples containing organisms identified as *N*. *meningitidis*, *N*. *sicca*, *N*. *flavescens* and *N*. *subflava* were tested. Due to its sensitivity and specificity, the Versant assay represents a good choice for the diagnosis of chlamydial and/or gonococcal infections not only in genito-urinary samples, but also on rectal and pharyngeal swabs.

## Introduction

*Chlamydia trachomatis* and *Neisseria gonorrhoeae* infections are the most common bacterial sexually transmitted infections (STIs) worldwide, representing important problems of public health [1].

Anal and pharyngeal intercourse has been increasingly recognized as a component of the sexual repertoire of many couples, leading to the creation of selective high-risk networks for STIs transmission. Kent *et al*. reported that 53% of chlamydial infections and 64% of gonococcal infections in a selected population of men having sex with men (MSM) were localized at non-urethral sites [2], while Jones and colleagues found that 6.4% of women with chlamydial infection harboured the microorganism only at rectal site [3].

Since chlamydial and gonococcal extra-genital localizations could be asymptomatic or characterized by non-specific symptoms, they can remain unnoticed in many cases and may act as important reservoirs for further transmission [2,4–5]. It follows that an accurate diagnosis for extra-genital *C*. *trachomatis* and *N*. *gonorrhoeae* infections, reliant upon highly sensitive and specific tests, is critical to STI control efforts.

Nucleic acids amplification techniques (NAATs) have become the tests of choice for screening rectal and pharyngeal chlamydial and gonococcal infections [6–9].

Nevertheless, at present no commercial NAATs have been approved for use on pharyngeal and rectal specimens by the US FDA or have received the conformity CE marking for the diagnosis of chlamydial and gonococcal infections [1,8]. For that reason, in the last years many laboratories have conducted their internal validation studies in order to provide results for the clinical management [10].

One of the most serious deterrents for the use of NAATs on pharyngeal and rectal swabs is the possibility of false *N*. *gonorrhoeae* positive results due to cross-reaction with non-gonococcal *Neisseria* species, frequently found in the oral cavity, oropharynx and rectum [11, 12].

The Versant CT/GC DNA 1.0 assay (kPCR) (Siemens Healthcare Diagnostics, Tarrytown, NY, USA), a duplex real-time PCR-based assay for the simultaneous detection of *C*. *trachomatis* and *N*. *gonorrhoeae*, has been routinely used in the Microbiology Laboratory of St. Orsola University Hospital, Bologna, since 2011 [13]. After a preliminary internal validation study (unpublished data) we decided to use this assay also for testing extra-genital specimens, finding several chlamydial and gonococcal cases, in particular in a high risk population attending the University Hospital STI center [14,15]. Besides the good performance showed by the Versant assay in clinical setting, no analytical studies about the limit of detection for *C*. *trachomatis* and *N*. *gonorrhoeae* of this commercial method on extra-genital samples have ever been published.

Therefore, the aim of the present study was to assess the analytical sensitivities of the Versant CT/GC assay in comparison with two in-house PCR assays on rectal and pharyngeal swabs. Moreover, an evaluation about the suitability for the commercial assay with two widely used swab collection devices (E-Swab and eNAT, Copan, Brescia, Italy) was carried out, as well as the specificity assessment of the Versant assay, in order to exclude problems of false positive results due to the presence of non-gonococcal *Neisseria* species in rectal and pharyngeal suspensions.

## Materials and Methods

### Molecular assays amplification and detection

The Versant molecular system consists of a sample preparation module designed for automated sample preparation and an amplification/detection module designed for real-time (or kinetic: k) PCR and detection. *C*. *trachomatis*-specific PCR primers and probe were designed to target the GenBank nucleic acid sequence of the 7.5-kb cryptic plasmid. *N*. *gonorrhoeae*-specific PCR primers and probe were designed to target the nucleic acid sequence of the 7 to 8 copies of *pivNG* gene loci.

The Versant CT/GC assay was run on the Versant kPCR Molecular system [16]. After the DNA extraction and elution steps, remaining eluted specimens were further processed for in-house PCR assays, as follows. For *C*. *trachomatis* detection, a semi-nested PCR targeting *omp1* gene was performed [13,17]. The first product of 1033 base pairs (bp) was amplified using the following paired primers: SERO1A (5’-ATGAAAAAACTCTGAAATCGG-3’) and SERO2A (5’-TTTCTAGATCTTCATTCTTGTT-3’). In the second PCR step, amplifying a 978 bp fragment, primers SERO2A and PCTM3 (5’-TCCTTGCAAGCTCTGCCTGTGGGGAATCCT-3’) were used.

A PCR assay targeting a region of 132 bp of *porA* pseudogene was performed for the detection of *N*. *gonorrhoeae*, using papF (5’-CGGTTTCCGTGCGTTACGA-3’) and papR (5’-CTGGTTTCATCTGATTACTTTCCA-3’) as primer pair [18,19]. The amplified products were visualized after electrophoresis in agarose gel by ethidium bromide staining.

### Analytical sensitivity of the Versant assay and in-house PCR assays

Limits of detection (LODs) of commercial and in-house PCR methods on rectal and pharyngeal samples were determined by analyzing in triplicate a panel set with dilutions of CTB13-06 and NG13-01, two Quality Controls for Molecular Diagnostics belonging to the panels of QCMD 2013 *C*. *trachomatis* DNA EQA Programme B and QCMD 2013 *N*. *gonorrhoeae* DNA EQA Programme, respectively (www.QCMD.org). The LOD was defined as the lowest concentration to give a reproducible positive result.

As a rectal matrix, a mix of ten negative rectal samples, collected with E-Swab and eNAT devices, respectively, was used. Equally, as a pharyngeal matrix, a mix of ten negative pharyngeal samples, collected with E-Swab and eNAT devices, respectively, was used. Negative rectal and pharyngeal samples were self-collected from healthy volunteers reporting no unsafe sexual intercourse and who had previously been screened for chlamydia and gonococcal genital infections. In order to limit extraction failure problems due to the excessive foaming, the rectal and pharyngeal E-Swab and eNAT suspensions were prepared as follows: the tubes were vortexed for about 30 seconds, then the swabs were removed and thrown away. The suspensions were refrigerated at 4°C until the amplification and re-vortexed prior the loading on the Versant sample preparation module.

The rectal and pharyngeal E-Swab and eNAT suspensions were spiked with CTB13-06 and NG13-01 dilutions, as follows: CTB13-06, containing 10000 copies/ml, was diluted in order to obtain five different rectal or pharyngeal specimens, with a final *C*. *trachomatis* content ranging between 10^2^ to 10^-1^ copies/ml (specifically: 100, 50, 10, 5 and 1 copies/ml). Similarly, NG13-01, containing 10000 copies/ml, was diluted and the mock specimens had *N*. *gonorrhoeae* DNA values ranging between 10^2^ to 10^-1^ copies/ml (specifically: 100, 10, 5, 1 and 0.1 copies/ml).

As reference blank samples, a mix of 10 blank E-Swab liquid sample suspensions and a mix of 10 blank eNAT liquid sample suspensions were also spiked with CTB13-06 and NG13-01 dilutions.

### Ethics Statement

A written informed consent was obtained from all of the volunteers and the study was approved by the S. Orsola-Malpighi institutional review board (CE 52/2014/U/Tess number).

### Specificity assessment

In order to exclude problems of false positive results due to the presence of non-gonococcal *Neisseria* species, twenty E-Swabspreviously found positive for other than gonococcal neisseriae were tested by both the Versant and *porA* PCR assays. In particular, the specimens included pharyngeal E-Swabs obtained from patients suffering from pharyngitis and rectal E-Swab submitted for carbapenem-resistant *Enterobacteriaceae* screening. Neisseriae were identified during routine diagnostic procedures at species level by matrix-assisted laser desorption/ionization time of flight mass spectrometry (MALDI-TOF MS; Bruker Daltonik GmbH, Leipzig, Germany) and classified as *N*. *meningitidis* (n = 5), *N*. *sicca* (n = 5), *N*. *flavescens* (n = 5), and *N*. *subflava* (n = 5).

### Statistical analysis

Statistical analyses were performed by using a two-tailed unpaired Student’s *t* test for continuous data (GraphPad Prism version 5.02 for Windows, GraphPad Software, San Diego California USA, www.graphpad.com). A *P* value <0.05 was considered significant.

## Results

**Fig 1:**
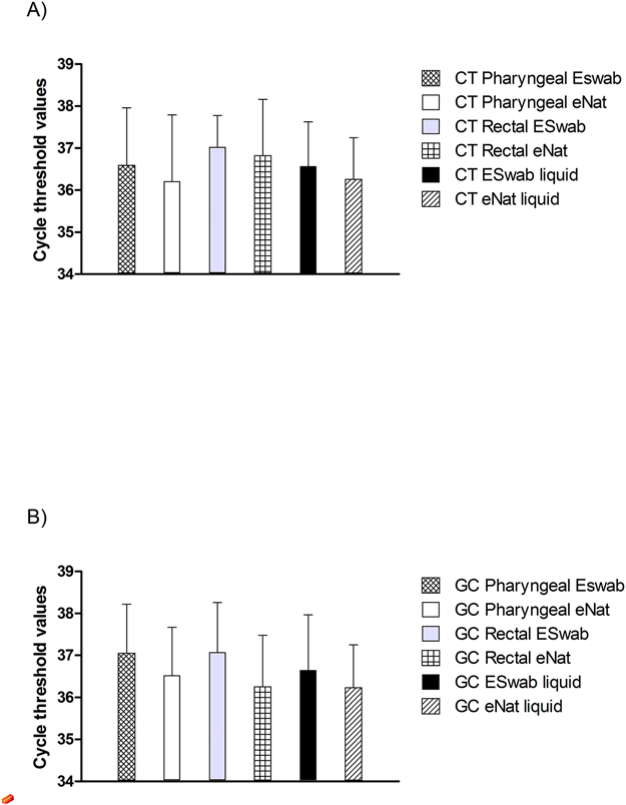
Cycle threshold values. Cycle threshold values of mock and reference specimens obtained by the Versant CT/GC DNA 1.0 Assay, when suspensions containing 10 copies/ml of *C*. *trachomatis* (Part A) and 1.0 copies/ml of *N*. *gonorrhoeae* (Part B) were tested. Error bars represent standard deviations (SD).

The *C*. *trachomatis* LOD of Versant CT/GC assay on mock specimens was 10 copies/ml. The cycle threshold values were similar, with no significant differences, when the two collection devices were used and/or the two matrices were compared. Moreover, these results were comparable to those obtained by reference blank specimens’ analyses, being again the *C*. *trachomatis* LOD 10 copies/ml (Fig. 1A).

Similarly, the *N*. *gonorrhoeae* LOD of the Versant CT/GC assay on mock specimens was extremely low (1.0 copies/ml), with no significant differences regarding the collection devices used or the sites of collection. Again, when reference samples were tested, the LOD and cycle threshold values were comparable to those obtained by mock specimens’ analyses (Fig. 1B).

Finally, it is also noteworthy to underline that the Versant assay internal control did not show significant differences involving the collection devices or the matrices used (data not shown).

The chlamydia LOD of the in-house *omp1* PCR assay was higher than that of the commercial method (50 copies/ml) regardless of the collection devices used or the sites of collection.

Similarly, the gonococcus LOD of the *porA* PCR assay was higher than that of the Versant assay, with no significant differences for the matrices or the collection devices used (5 copies/ml).

Finally, no clinical swabs identified as positive for non-gonococcal *Neisseria* species by culture methods during routine diagnostic procedures scored reactive when analyzed by both Versant and *porA* assays.

## Discussion

Nowadays, a substantial proportion of the population engages in oral and anal sexual activities, leading to high *C*. *trachomatis* and *N*. *gonorrhoeae* infection rates at these sites [20]. It follows that recent guidelines suggest screening of rectal and pharyngeal sites in addition to genital specimens, based on a patient’s sexual history [1,21].

As highlighted by Peters *et al*, the strategy of sexual-history based screening of multiple anatomic sites for *C*. *trachomatis* and *N*. *gonorrhoeae* in MSM population is a valid and useful guideline which is to be preferred over a symptom-based screening protocol [22].

There may be issues with the specificity of NAATs for *N*. *gonorrhoeae* performed on pharyngeal or rectal samples due to the potential presence of non-gonococcal *Neisseria* species [11,12]. In the present study, the high specificity of the Versant assay was confirmed by the lack of cross-reactivity with non-gonococcal *Neisseria* species identified in the pharyngeal and rectal swabs during routine diagnostic procedures. Unfortunately, since *N*. *gonorrhoeae* exhibits a natural competence for transformation, and there is quite frequent genetic exchange with other *Neisseria* species [11], it cannot be completely excluded the possibility of false-positive results when applied to rectal and pharyngeal specimens. It should be indeed remembered that Bongaerts [23] found that a QCMD sample belonging to the QCMD 2010 *N*. *gonorrhoeae* DNA EQA Programme (NG10-02), containing *N*. *cinerea*, showed a positive cycle threshold value when tested by the Versant assay. Anyway, these authors underlined that the amplification curve was very strange and non-logarithmic, with an unexpectedly low final fluorescence value, so it was quite easy to recognize it as a false-positive signal.

We had the chance of experiencing the same phenomenon when testing other QCMD samples prepared with this particular *N*. *cinerea* strain (namely NG11-03 and NG12-03, belonging to the QCMD 2011 and QCMD 2012 *N*. *gonorrhoeae* DNA EQA Programmes, respectively).

The manufacturer, informed about these false-positive signals, found by sequencing that the cross-reactivity was due to a part of the *pivNG* gene included in this particular *N*. *cinerea* strain by recombination.

A second important deterrent for the wide use of NAATs on pharyngeal and rectal swabs is due to the possible false-negative results caused by the presence of inhibitory substances [24].

In the present study, we were able to exclude the presence of false negative results due to a target inhibition, as the observed Versant assay LODs were extremely low and never affected by the site of collection. Similarly, the two in-house PCR assays, even if less sensitive than the commercial test, did not show any inhibition.

Finally, we excluded any interference due to the different liquid components of E-Swab and eNAT mediums, demonstrating that both of the devices are valid options for the collection of secretions to be tested by the Versant assay: as a matter of fact, when the spiked E-Swab and eNAT suspensions were tested, the cycle threshold values of the internal control, chlamydia and gonococcus were very similar, with no significant differences between the two collection devices. Moreover, following the suggested method above reported, we did not encounter any extraction failure problems due to the excessive foaming of the liquid suspensions.

Until molecular methods for the detection of *N*. *gonorrhoeae* gene sequences that confer resistance are validated for routine laboratory use, testing by both culture and PCR is advisable to continue surveillance of antibiotic susceptibility [25]. It is noteworthy that the Versant assay allows testing of specimens collected by E-Swab, in contrast to other commercial NAATs using high-salt collection mediums that are not able to maintain viable organisms for culture [26]. This characteristic is particularly intriguing since it is possible to use the same sample for both PCR and culture.

In conclusion, NAATs that have become the reference method for *C*. *trachomatis* and *N*. *gonorrhoeae* detection both on urogenital and extra-genital specimens, have also been shown to be reliable, fully-automated, and high-throughput methods [20,27–28].

In this context, the Versant CT/GC 1.0 assay can be a smart choice, since the time to result of less than 5 h allows the processing of 94 specimens plus 2 controls, with only a few minutes of hands on time [16]. Furthermore, it has been proven a sensitive and specific method even on rectal and pharyngeal samples. Anyway, since genetic exchange can happen for *Neisseria* species, a second NAAT targeting another sequence would be advisable, in particular in low-prevalence populations, when the PPV (Positive Predictive Value) is lower. For this purpose, the proposed in-house *PorA* assay can be a cost-effective alternative, being relatively inexpensive and targeting a gene that demonstrated to be less involved in genetic recombination than others [11].
